# Supplementary material for: The role of civil society organizations (CSOs) in the COVID-19 response across the Global South: A multinational, qualitative study
Source: PLOS Glob Public Health. 2023 Sep 14;3(9):e0002341. doi: 10.1371/journal.pgph.0002341 (PMC10501645; doi:10.1371/journal.pgph.0002341)
Supplement: S1 Appendix — (DOCX) [file pgph.0002341.s001.docx]

# **S1 Appendix:** Profiles of Sampled Organizations

## **Mexico**

**Alternativas y Capacidades, A.C. [Preliminary interview]**

Alternativas y Capacidades is an organization focused on strengthening civil society. It was born in 2004, operates nationally and has a permanent staff of 12 people. Its goal is to provide a platform to promote the work of CSOs in the country, connect CSOs to donors and provide professionalization workshops to small and medium CSOs. It also releases reports on the state of CSOs and the impact of budget initiatives by the government. To respond to the pandemic, it created a media campaign to make the work of CSOs visible (#AsíRespondimos) and did a survey to assess the impact of the pandemic and the type of responses from CSOs.

**Centro de Derechos Humanos de la Montaña (Tlachinollan)**

Tlachinollan was created in 1993 to provide support to victims of military violence in the mountain region of the south of Guerrero. Since then, it provides legal counseling, direct relief and services to the communities living in the area, who are mostly agrarian domestic migrants, Indigenous Peoples (especially Na savi, Me’phaa, Nauas and Nn’anncue), families of disappeared people and feminicide victims, and survivors of sexual assault. It receives no volunteers because of violence in the municipality where the offices are (Tlapa de Comonfort in Guerrero). Tlachinollan distributed food and sanitary kits to community members, monitored symptoms and conditioned its shelter for internal migrants. It participated in advocacy campaigns on the need for food in the region, and on the increase in violence against women during the pandemic. It also lobbied with the government to facilitate the repatriation of remains from the US who had died of Covid-19 back to the region.

**Centro de Derechos Humanos Fray Matías de Córdova**

The Human Rights Center Fray Matías de Córdova (CDH Fray Matías) was created in 1994 in the border city of Tapachula, Chiapas. It works on human rights defense of migrants and refugees, providing legal counsel, accompaniment in migration processes (from counseling to psychosocial help) and information workshops. It has an area of research and advocacy. CDH Fray Matías participated in networks to monitor and diffuse information on the conditions of migrants and refugees during the pandemic. It carried out legal consultations and accompaniment in person and over telephone.

**El Caracol**

Organization working with the homeless population of Mexico City since 1994. The headquarters have a dining area and showers. El Caracol provides education modules for homeless children, programs about death prevention amongst the population and advocacy. It also provides judicial and housing assistance. It is composed of a core team of 10 people and volunteers. El Caracol created brigades to distribute sanitary kits, information about the pandemic and food packages. It also created a campaign to promote vaccination amongst homeless people and advocated for the government to allow people without addresses to register for the vaccine.

**Espacio Migrante**

Espacio Migrante is a binational community-based organization working in Tijuana and San Diego since 2012. It supports refugees, asylum seekers and migrants. Its main areas of work are education, human rights, cultural exchange, orientation and social work, community organization, and advocacy. Espacio Migrante delivered food and electronic cash cards. It modified the policies at their shelter to mitigate the spread of the virus and organized virtual workshops on the new migration restrictions that US and Mexico put in place because of the pandemic.

**Frente Nacional de Trabajadores de la Salud (FNTS)**

The “National Front of Healthcare Workers” (Frente Nacional de Trabajadores de la Salud, FNTS) is a social movement created in April 2020 gathering eight organizations working for labor rights of healthcare workers. These organizations include the National Assembly of Nurses, a National Doctor’s Movement, a Platform of Sub-hired Workers in the Medical Sector, and an association of chemists working in the health sector. It spans private and public HCW. The Front organized its first protest in May 2020. Since then, it has participated and organized protests of HCW in different states. It has connected HCW through WhatsApp and social media, diffusing information on the impact of the pandemic on HCW and the precarious work conditions. Its leaders have done interviews in the media and held meetings with public officials.

**Grupo de Información en Reproducción Elegida (GIRE)**

Information on Reproductive Choice (*Grupo de Información en Reproducción Elegida*) (GIRE) is a feminist organization founded in 1992 that advocates for multiple aspects of reproductive justice, such as abortion, contraception, obstetric violence, maternal mortality, assisted reproduction and work-life balance. It does so through advocacy, communication campaigns, research, and legal casework. GIRE led the Gender Observatory on the impact of COVID-19 and coordinated national advocacy efforts to highlight the effects of the pandemic on women. It is a network node that connects small and medium-size organizations.

**JADE Propuestas Sociales y Alternativas al Desarrollo**

JADE was created in 2018 as an organization focused on human rights and alternative development in Yucatán. It has three main areas: research, advocacy and social projects. It works with municipal bureaucrats in projects of “open governance” and advocates for regulations for domestic workers. JADE created a diagnosis on conditions of domestic workers during the pandemic, participated in advocacy networks and published reports on this issue.

**Other Dreams en Acción (ODA)**

ODA is a grassroots organization created in 2016 by and for deported and returned people that were born in Mexico, grew up or lived many years undocumented in the United States, and are now back in Mexico because they returned or were deported. It provides accompaniment and material support to individuals and their families to help them navigate their arrival in Mexico City. It has a network of ‘solidarity professionals’ that works with the returned or deported migrants and provides in person and virtual workshops with advice and community building activities. ODA gave food packages, resources to pay bills and housing aid to about 160 families and individuals. It also conducted workshops and virtual activities.

## **Kenya**

**Homeless of Nairobi**

Homeless of Nairobi is an organization that provides food assistance to homeless individuals in Nairobi’s Deep Sea slum through its daily street feeding program. Now a registered organization formally called the Odijo Foundation, Homeless of Nairobi began as a Facebook initiative (styled after “Humans of New York”) that brought attention to homeless individuals in Nairobi. The initiative has garnered significant attention within Kenya and abroad and has been covered by both local media houses as well as international ones including *The Guardian,* DW, and the BBC. While enjoying this broader renown, the organization continues to have a strong local character, relying primarily on nearby affluent communities for donations, and serving the local Deep Sea slum. Currently, Homeless of Nairobi’s work extends to skills training for out-of-school youth and providing accommodation through its children’s home.

**Kenya Medical Practitioners and Dentists Union (KMPDU)**

The KMPDU, which represents around 5,500 healthcare professionals, is the largest trade union for workers in the Kenyan health sector. The union began as an informal association organized by medical students and young doctors in 2009 via Facebook and was registered as a formal trade union in 2011.^^[[1]](#footnote-1)^^ Apart from its work on labor issues, KMPDU is involved in health advocacy and policy. In the context of the COVID-19 pandemic, KMPDU was the foremost CSO in Kenya and received heavy media coverage. The union was vocal in its critique of the unsafe working conditions of medical personnel and provided information on the pandemic to the public. KMPDU’s work in the pandemic also included working with the government as part of a taskforce established to address the capacity of the healthcare system, particularly its human resources, in the pandemic.

**Lifesong Kenya**

Lifesong Kenya is a registered community-based organization that works with youth (mostly boys) at risk of incarceration and those in prison. Founded in 2012, the organization’s activities include supporting youth in police custody and advocating for alternative punishment (to imprisonment); facilitating reconciliation between sentenced youth and their families and the aggrieved parties; providing psychosocial support and skills training to imprisoned youth in their prison visit program; and providing transitional housing for at-risk youth. Lifesong Kenya was included as it represents CSOs whose work was severely disrupted by pandemic lockdown measures, but nonetheless pivoted to other public action activities.

**MUHURI**

MUHURI (Muslims for Human Rights) is a formally registered NGO founded in 1997 and based in Mombasa (with an office in Garissa, in Northern Kenya). While primarily focused on historically marginalized groups in the Coast and Northern regions, MUHURI also addresses human rights violations at the national level. MUHURI is included to represent the human rights sector, and for its active response to the human rights violations committed by the state in enforcing lockdown measures and vaccine mandates. The primary strategies employed by MUHURI in this work were filing court petitions and in media campaigns.

**Safe Hands Kenya**

Safe Hands Kenya is a network of more than 40 organizations – local CBOs and NGOs, and private local and multinational corporations - that was formed to provide preventative sanitation services in densely-populated urban informal settlements. These services included providing handwashing stations, masks, as well as a public awareness campaign “TibaNiSisi” (“We are the Cure”). Safe Hands Kenya and Shikilia (below) share some of their founding member organizations with Safe Hands Kenya designed to target the first-order public health nature of the pandemic, and Shikilia aimed at the second-order economic consequences of the pandemic.

**Shikilia**

Shikilia is a coalition of private firms (mostly in technology) and NGOs that provided cash transfers to individuals whose incomes were affected by the pandemic. The network came together early in the pandemic, in March 2020, anticipating the economic impact the pandemic would have on low-income households, particularly those in informal urban settlements. Shikilia directed donors globally, both corporate and individual, to GiveDirectly which is registered in Kenya and has experience making cash-transfer payments. Roughly USD 5 million, which would support around 50,000 households, was raised. Cash payments of Ksh. 3000 (USD 30) for 3 months was disbursed to individuals identified as vulnerable (that is, the beneficiaries did not sign up).

**Touch a Soul**

Touch a Soul is an informal grassroots CBO located in the low-income urban settlement Mukuru kwa Njenga in Nairobi. The organization is constituted by around 30 members who are residents of Mukuru kwa Njenga. Touch a Soul’s work addresses general social welfare challenges in their area. Through a regular databasing effort, Touch a Soul identifies the urgent needs of especially indigent households and mobilizes resources to meet these needs. Key areas of work are food provision, material and social support to a local children’s home, and educational support.

**United Sisters of Nairobi**

The USN is a group of middle- and upper-class Muslim women that has been organizing since 2017. The group consists of almost 200 members based primarily in Nairobi, with a few members based in Mombasa and abroad. The organization is not formally registered. Monthly membership fees (Ksh. 1000/USD10) are remitted through M-Pesa, the mobile cash transfer software. The USN primarily targets marginalized communities in remote areas across the country and provides them with long-term welfare projects, mostly clean water, through digging boreholes. Other activities include donating basic provisions, as well as building mosques and schools in marginalized Muslim communities. I include USN in my sample of CSOs in Kenya because such informal voluntary associations that operate on media platforms are prevalent in Kenya where there has long been a history of *harambee* - community self-help initiatives.

##

## **South Africa**

**Abahlali baseMjondolo (AbM)**

This is a large grassroots social movement of people living informal settlements, largely in Ethekwini, the third most populous metropolitan municipality in the country. The movement’s isiZulu name means in English, “people who live in shacks.” AbM has been subject to frequent violent attacks by the Ethekwini Metrop olitan Municipal government in its stronghold settlements, most notably Kennedy Road. This has led its leadership to go into hiding at a few different points over the past decade. AbM has been largely skeptical of engaging in formal politics, but notably endorsed the opposition Democratic Alliance in the 2014 national elections as a protest against the ANC. It claims to have an audited membership of approximately 100,000, which would make it the largest non-party-aligned grassroots membership organization in the country. It is largely focused on resisting evictions and advocating for the provision of housing and upgrading of informal settlements.

**C19 People’s Coalition**

This is a coalition of a number of both existing and new CSOs that emerged in the very early days of the pandemic. The goal was to create a forum for CSOs to network, provide immediate aid, and create a platform for joint civil society inputs and activism related to the emergency policy-making process. This included professional NGO activists and organizations, as well as grassroots and community-based formations. The coalition was established in a multi-layer organizational form, with national, provincial, and local coordinating committees. By design, this was supposed to enable decentralized decision-making. The coalition projected significant strength in the early days of the pandemic. It was soon hamstrung by internal conflicts that created significant variability in effectiveness and organizational sustainability across various parts of the coalition.

**Development Action Group (DAG)**

DAG is one of the oldest urban development NGOs in South Africa, based in Cape Town. It serves as a support professional NGO to a range of community-based organizations throughout the city’s metropolitan area, and during the pandemic increased its reach to more rural communities in the Western Cape province. It also has strong relationships with city government and has a long history of inputting into policy-making processes.

**Health Justice Initiative**

This was a new organization that started during the pandemic, largely focused on domestic public health policy and international vaccine equity. It is founded and led by a well-known social impact lawyer who was active as part of the Treatment Action Campaign’s work advocating for access to antiretroviral drugs for those with HIV. It has been active on public health policy, linking to key figures in national government, as well as part of the main international networks advocating for access to coronavirus vaccines in poor and middle-income countries, including on issues of opening up intellectual property.

**Institute for Economic Justice**

This is a relatively new organization, that was founded shortly before the start of the pandemic. The goal of the organization is to serve as a hub for labor-oriented heterodox economic policy. Prior to the pandemic, its first major project had been to develop some of the key research inputs that influenced the national government’s deliberations over policy for a national minimum wage. During the pandemic, it used connections to the national treasury and COSATU to influence and convene policy debate about key emergency fiscal measures in economic policy.

**Pay The Grants Campaign**

This is a campaign that has included professional researchers and grassroots organizations, focused on ensuring receipt of an emergency grant instituted by the government as a response to the pandemic. Much of this work was initially coordinated through the C19 Coalition, initially to pressure for the establishment of this grant in the first place.

**South African Democratic Teachers Union**

This is one of the largest unions in COSATU and represents teachers throughout the country. It counts over 250,000 members. It was involved in coordinating remote learning and influencing policy to ensure safe working environment for teachers across rural and urban South Africa.

**South African Labor and Development Research Unit**

This is a longstanding economic research unit at University of Cape Town, which took on a significant role in both influencing policy formulation in the presidency and coordinating key conversations for continuing policy in the National Treasury.

**Seriti Institute**

This is a service delivery NGO that operates across Gauteng. Historicaly, Seriti had implemented grants from national and provincial government, largely around food aid. However, shortly before the pandemic it had lost a number of key government contracts due to cancellation of programs it had helped administer. During the pandemic, Seriti got involved in emergency food provision, largely through encounters with new actors as part of the C19 Coalition.

**Tembelihle Crisis Committee**

This is a leadership formation in the informal settlement of Tembelihle in the southern part of Johannesburg. It has fought eviction of the community for many years, and advocated for the upgrading of its community. Its stance towards government has largely been oppositional and it is distant from ANC formations. Its leaders helped coordination the Community Working Group of the C19 Coalition, which led it to pursue new efforts at city-wide coordination of informal settlement communities, a longstanding challenge of housing and urban activism in Johannesburg.

## **India**

**Bro.Siga Animation Centre**

This is an NGO in Chennai working toward the empowerment of slum and rural poor. It mainly works with children and their communities in Vyasarpadi, an area in North Chennai which is primarily inhabited by people belonging to the lower economic strata of society. Bro.Siga Animation Center believes that its work is aiming at the empowerment of the people to create a self-sustained community. Bro.Siga Animation Center implements various initiatives seeking to promote children and youth development, education and children’s rights.

**Arunodhaya**

This is a charitable trust which works on children’s right, well-being and protection in the northern part of Chennai. The NGO has built a social movement which has helped eliminate child labor in the city and it continuously works on various programs to prevent children’s exploitation, enhance their rights and promote a more dignified childhood. In addition to running various programs for children and strengthening community organizations working on children’s protection, Arunodhaya also seeks to effect policy changes and social justice through research, documentation campaigns, and networking.

**Center for Youth and Social Development (CYSD)**

CYSD is a non-government and non-profit organization established in 1982, working to improve the quality of lives of tribal, rural and urban poor in Odisha, with a primary focus to eradicate extreme poverty and hunger, ensuring social inclusion and justice, good governance and citizen’s right. Helping communities identify and initiate development measures; providing training and other capacity-building support to pro-poor organizations and individuals; and carrying out research and advocacy in favour of the under privileged people especially the tribal. Started the org 40 years ago because they wanted to engage youth in social development activities. Main lines of work: 1) Participatory governance; 2) Focus on tribal people, indigenous communities, sustainable livelihood programs, basic services, access to those, improving access to those; 3) Disaster management, cyclones, disaster response organization; 4) Work with a large network of organizations – networking is a strategy to reach beneficiaries, scaling up pilots.

**Trans Rights Now Collective**

This is a Dalit- Bahujan- and Adivasi-centred collective of trans folx working towards greater trans visibility and opportunity in education, the workplace, and politics. This social movement was founded by a local transgender activist from Tamil Nadu state. who has campaigned and worked on various issues advancing rights of transgender community in India, including a passing of the court order that gives an opportunity for transgenders in the Tamil Nadu public service recruitment examination.

**Hemkunt Foundation**

This small Gurugram, Delhi-based organization founded in 2010 started with a small project of running free schools in 3 districts of Delhi and gradually expanded to other programming focusing on education, sanitation, and economic upliftment of the poor, and most recently established itself as a major relief provided during the Covid-19 pandemic.

**Information and Resource Centre for the Deprived Urban Communities (IRCDUC)**

The Chennai-based organization seeks to enhance the capacities of communities, facilitate community-led initiatives, disseminate information about laws and policies, and undertake policy research related to land and housing rights of the deprived urban communities. It was started in 2011, and was initially a group based only on volunteers who worked on housing rights in Chennai, and later expanded to Madurai and Coimbatore. The IRCDUC focuses on three activities. First, as a community centric information hub, the IRCDUC translate policy documents to local Tamil language. Second, they share this information in appropriate manner with communities and enhance their capacity to take action with the help of researchers and citizens; IRCDUC acts as a facilitator between citizens and government. They obtain citizen feedback and information and share it with the government and policymakers. Third, they create “information centres” about settlements by training 4-5 women in a particular settlement on to how to get entitlements and how to identify domestic work, domestic violence, and child sexual abuse. These volunteers are given basic human rights training so that they are able to take up the issues and take it further in the community. IRCDUC is also a member of the state level monitoring committee for homeless shelters.

**Mahila Kisan Adhikaar Manch (Makaam), (Forum for Rights of Women Farmers)**

MAKAAM is an Alliance of networks, campaigns, movements, organizations, people’s collectives and individuals from across 24 states in India, that advocates for the Right to Livelihoods of Women Farmers, particularly dalits, adivasis, single women, differently abled and displaced. MAKAAM also focuses on the issues of women from farm suicide affected households, women sugarcane cutters, women and land rights, and forest rights.

**Mercy Mission**, **Bangalore**

A coalition of NGOs launched the ‘Mercy Mission’ in Bangalore soon after the lockdown announcement, on March 29, 2021. The group had been planning for a crisis response system even before the pandemic hit (already in December 2019). The leader of the group was a founder of NGO called “Smile”. The coalition was concerned with setting up an emergency response system and was already thinking about how they would act collectively in a pandemic or another kind of crisis. Their goal was to be centralized but have a system of dispersed local hubs and thus the group’s efforts weres limited to Bangalore. All NGOs divided areas they would be working in not to duplicate their efforts, and the whole system was set up in a couple of days.

**Rashtriya Swayamsevak Sangh (RSS)**

RSS is a Hindu nationalist organization. It does not have a formal membership. Those who attend the RSS Shakhas are called Swayamsevaks and any Hindu male can become a Swayamsevak (no women can become an RSS member). Shakha is a daily gathering of swayamsevaks of different age groups at a predefined meeting place or ground for one hour. The daily routine programs include physical exercises, singing patriotic chorus, group discussions on varied range of subjects and a prayer for our motherland. RSS believes that India belongs to the Hindus. The RSS perceives Hindu as a term that defines the national identity of the people living in this country, and not a religious or sectarian identity, rather “a way of life.” Primary activity of RSS in Pune before Covid-19 was holding Shakhas. The RSS is one of the world’s largest non-government associations with approximately 1.5-2 million members that participate in its daily meetings. Shakhaas take place in approximately 36,000 locations across the country. These shakhas for the “structural foundation” of the RSS and are also used as nodes for welfare provisioning (Andersen and Damle 2018)

**Sangtin Kisan Mazdoor Sangathan (sangtin)**

This is a people’s movement in Sitapur District of Uttar Pradesh in India. It emerged from Sangtin, a group formed by rural women to enable them to shape the processes of development at all levels. The SKMS saathis or members are mainly marginal farmers or landless laborers in rural Sitapur. More than 90 percent of the approximately 8000 voluntary saathis identify as Dalit, with women and men being equally active in the Sangathan. The overall planning is done by a 45-member core group. Working through collective action, SKMS has led many struggles in the District, e.g., reviving and repairing canals, ensuring employment under the Mahatma Gandhi National Rural Employment Guarantee Act (MGNREGA), obtaining pensions for the economically vulnerable, and realizing compensation during natural disasters. SKMS saathis were the first in Uttar Pradesh to realize unemployment benefits under MGNREGA; in January 2009 almost 15 lakh rupees were paid to 826 families in Mishrikh and Pisawan blocks of Sitapur District. Since then, SKMS has expanded its work to health, agricultural, and livelihood issues and spread across half the blocks in the District. SKMS is active in various state-level and national rights-based campaigns and networks.

**Working People’s Charter (WPC)**

WPC is a national coalition of organizations working on issues related to informal labour in particular, and labour in general. It is an independent entity not affiliated to any organization, federation or political party. The WPC is open all organizations which are working with informal labour – in organizing, support, research, training, skilling etc. - irrespective of affiliation or sector, who are in broad agreement with the Working Peoples Charter.

## **Philippines**

**Agro-Eco Philippines**

Agro-Eco Philippines is a farmer-led national network-organization established in 2004. The organization provides services related to sustainable farming and capacity building programs for climate change resilience in agricultural areas across the country through its partnership with people’s organizations, local government units, non-government organizations, higher education institutions, and private sector.

**Angat Buhay/ Kaya Natin Movement for Good Governance and Ethical Leadership**

This organization started through a policy forum initiated by one of the country’s leading universities, Ateneo de Manila University, in collaboration with several local government officials known for their anti-graft and corruption initiatives in 2008. The organization later developed into a volunteer-led movement supported by the Vice President of the Philippines, Leonor “Leni” Robredo in collaboration with a network of CSOs and private sector organizations with the aim of mitigating the impacts of poverty and climate change among vulnerable sectors.

**Assistance and Cooperation for Community Resilience and Development**

Assistance and Cooperation for Community Resilience and Development, Inc (ACCORD) was established in 2010 as a non-profit organization with the aim of providing support for vulnerable communities affected by major disasters. Its core programs center on local capacity building to manage the impacts of poverty and promote sustainable human development.

**Coalition of Services of the Elderly**

The Coalition of Services of the Elderly, Inc. (COSE) was founded in 1989 by different NGOs and private organizations centered around working with older persons in the Philippines to promote their quality of life through access to health, financial, and psycho-social support. The organization collaborates with local government units and private sectors in their program development and implementation. In 2021, it has more than 100 affiliated organizations across the country.

**Community Pantry PH**

The Community Pantry PH is a social movement inspired by the Maginhawa Community Pantry that was informally launched by a local resident, Anna Patricia Non, at the peak of the implementation of the Enhance Community Quarantine resulting from a COVID surge which prohibited individuals from leaving their homes. Other villages have later on copied the “take what you need, give what you can” policy of the community pantry. The movement that is highly based online was created to sustain community pantries in the Philippines.

**DAMPA- Damayan ng Maralitang Pilipinong Apo**

DAMPA, Inc. was established in 1995 after the demand for support for basic services and social protection increased in urban poor areas as demolition campaigns were implemented in the country’s capital–Metro Manila. Apart from providing resources to address basic needs, the organization also engage in community organizing that aim to empower the communities to communicate pro-poor legislation with the government.

**HIV/AIDS Support House**

HASH was co-founded by Desi Andrew Ching and Michael P. De Guzman in 2014 along with other HIV-AIDS advocates who identified the gap in treatment support for people living with HIV (PLHIV). The organization actively collaborates with international organizations and local government units to deliver services such as testing, housing, and access to ART for their stakeholders. The organization is based in the country’s National Capital Region.

**Kythe Foundation Inc.**

Kythe Foundation Inc was founded in 1992 as a non-profit, non-stock organization with the mission of promoting the quality of life of children hospitalized for cancer and other chronic diseases. Through their program called Kythe Child Life Services, the organization links the children and their families to hospitals, donors, and volunteers. It now has 10 hospital affiliations across the country.

**Leyte Center for Development**

The Leyte Center for Development, Inc. (LCDE) was established in 1986 as a response to the negative socio-economic impacts of the Martial Law. Formerly known as Integrated Lay Workers’ Association (ILAW) Resouce Center (IRC), LCDE aimed at promoting community empowerment through evidence-based socio-economic services. After the onslaught of Super Typhoon Haiyan, the organization took an active role in equipping vulnerable communities towards disaster response and mitigation through sustainable development. Initially just based in Leyte, the organization now has offices in other parts of the region.

**Philippine Rural Reconstruction Movement (PRRM)**

One of the oldest civil society organizations in the Philippines, PRRM has been working towards sustainable and equitable development in the rural areas of the country since 1952. Using a fourfold approach to development, the organization implements programs covering the areas of education, livelihood, health, and self-governance. With satellite offices across the country, it utilizes a grounded data approach in promoting policy development with government offices. Its most successful contribution to grassroot democracy is the institutionalization of the Barangay Council–the small political unit in the country mandated to govern villages.

**RH Forum Incorporated**

Established in 2004, the Forum for Family Planning and Development (also referred to as RH Forum) has been promoting reproductive health in parts of the country that have been identified to have issues on maternal, neonatal, child health and nutrition, adolescent and youth reproductive health, STI/HIV AIDS control, and gender-based violence. Through capacity building programs, RH Forum engaged local government units in developing policies and programs that could address these issues. They have coordinators in vulnerable areas and have actively collaborated with government actors to craft policies on reproductive health and family planning.

**Rise Against Hunger Philippines**

Born out of the demand for food intervention in the aftermath of Typhoon Haiyan, Rise Against Hunger Philippines aimed at immediately deploying food packages to the survivors of the disaster. In 2014, the constant demand for food interventions coming from communities affected by natural disasters necessitated the creation of the local office of the international organization Stop Hunger Now which later rebranded as Rise Against Hunger. With international funding and national registration, the organization has successfully delivered almost 8 million packed meals to vulnerable households. It is the first food bank in the country.

**Tahanan Outreach Projects and Services**

Founded in 1969 by Justice Corazon Juliano Agrava, TOPS aims at promoting human development through a comprehensive and integrated approach among the youth who are in a vulnerable or crisis setting. With programs spanning from education to food security, TOPS has been actively engaging children and youth towards capacity building that aim to disengage them from risky activities. Primarily funded by private individuals, the organization has focused its interventions in the National Capital Region.

1. Mwenda, Aruyaru Stanley. 2012. “From a Dream to a Resounding Reality: The Inception of a Doctors Union in Kenya.” *The Pan African Medical Journal* 11:16. [↑](#footnote-ref-1)
