# Supplementary material for: The role of civil society organizations (CSOs) in the COVID-19 response across the Global South: A multinational, qualitative study
Source: PLOS Glob Public Health. 2023 Sep 14;3(9):e0002341. doi: 10.1371/journal.pgph.0002341 (PMC10501645; doi:10.1371/journal.pgph.0002341)
Supplement: S2 Appendix — (DOCX) [file pgph.0002341.s002.docx]

Qualitative Agenda

**PROGRAMS AND SERVICES**

**Overarching Question**

What COVID related interventions has your organization been involved with?

**Sub Questions**

- Operations:
  - Did the organization’s pre-existing programs and services continue during the pandemic?
  - Will the pandemic change future operations, even after the pandemic recedes?
- Populations:
  - Have new populations been incorporated into the organization’s work as a result of the pandemic?
  - Did access to certain populations become more or less challenging?
  - How has the population served influenced the organization’s activities?
- Change over time and testing/vaccine rollout:
  - How have interventions changed, shifted, or evolved as the pandemic continued?
    - What were early interventions, vs. more sustained interventions as months went by?
  - How have organization’s programs and services been affected by testing?
  - Have programs changed with the distribution of vaccines?

**ORGANIZATIONAL CAPACITY AND STRUCTURE**

**Overarching Question**

How has the COVID-19 pandemic affected your organization’s structure?

**Sub Questions**

- How is the organization structured - traditional hierarchical or flat, formal or informal?
- How have the organization’s personnel reacted to the pandemic/made changes to their everyday work?
  - Are personnel operating in person or virtually? How has that affected the organization?
- Did the organization have to make changes to equipment, internet connections, work site, or personnel?
- Has the organization’s volunteering base increased or decreased with the pandemic? Has the composition of volunteers changed?
- Does the organization envision more permanent changes in their organizational structure/type, or will they try to ‘go back to normal’ after the pandemic recedes?

**COLLABORATIONS AND NETWORKS**

**Overarching Question**

How has the pandemic affected the organization’s collaborations and networks?

**Sub Questions**

- What networks of organizations do they belong to?
- Did the organization’s networks widen or decrease? Were new networks created in response to the pandemic? What will happen to these networks after the pandemic?
- Has there been network-wide collaborations/initiatives in response to COVID-19?
  - If so, what has been the nature of these initiatives?
  - How are interventions determined among those in the network? What is the decision-making and agenda-setting process?
  - What are the challenges and resolutions related to activities carried out with partner organization?
- Has the organization built upon these networks for advocacy?
  - - If so, what were the main axes of advocacy?
    - Who has been the main audience? (state/what level, population, other CSOs?)

**STRATEGIES**

**Overarching Question**

How did the organization decide on its main priorities and strategies for pandemic response?

**Sub Questions**

- How has the overarching nature of the pandemic been viewed locally by the organization? Like a natural hazard, a national crisis, state failure, etc.?
- Who did the organization identify as key constituents/targets in its response efforts? Why?
- How has the organization engaged constituents and what strategies are used to ensure representation?

**RELATION TO THE STATE**

**Overarching Question**

What is your organization’s relationship to state actors?

**Sub Questions**

- With what level of state actors does the organization interact with?
- What kind of support, if any, is the organization receiving from state actors? (financial, logistical, material)? Does the organization provide any direct support or resources to these state actors?
- Does the organization have political, religious, or professional affiliations with the state?
- How has the government’s approach to the pandemic impacted the organization? Has it hurt or helped the organization’s strategies, programs, services, and collaborations?
- Are there strategies or activities that the organization is using to pressure the state or make their voices heard? Repertoires of contention?

**FUNDS/BUDGET**

**Overarching Question**

How has the organization’s budget changed with the pandemic?

**Sub Questions**

- How have any budgetary shifts affected the type of programs that the organization is implementing during the pandemic?
- How has the organization’s funding landscape changed?
  - Did they gain or lose donors?
  - Did they receive funds from different sources?
  - Are resources more or less earmarked than before the pandemic?
- How has the organizations financial situation evolved with the pandemic?
  - Have there been changes between the early weeks of the pandemic vs. later in 2020 vs. now?
