# Supplementary material for: The role of civil society organizations (CSOs) in the COVID-19 response across the Global South: A multinational, qualitative study
Source: PLOS Glob Public Health. 2023 Sep 14;3(9):e0002341. doi: 10.1371/journal.pgph.0002341 (PMC10501645; doi:10.1371/journal.pgph.0002341)
Supplement: Attachment — Submitted filename: Reviewer Response_UPDATED.docx [file pgph.0002341.s003.docx]

**Response to Reviewers**

**Reviewer #1:** 

Thank you for taking the time to prepare and submit this manuscript. The topic is interesting and covers important gaps around how CSOs contributed during the COVID pandemic in LMICs. Below I detail several suggestions. In addition to these points, I suggest the authors look at the COREQ checklist and ensure their manuscript covers all points in the checklist prior to re-submitting the manuscript. One key area for improvement in the article is lack of illustrative quotations in the results section. The discussion section should also be revised, to ensure that the topics discussed and the conclusions drawn relate more directly to the results of the study. I suggest this article be revised and resubmitted. Once revised, this article stands to make an important contribution to the literature.

**Thank you for your constructive advice regarding the article. We have reviewed the COREQ checklist as recommended and made several updates throughout the article to ensure inclusion of the relevant checklist items. In particular, we have added illustrative quotes throughout the results section as recommended by the reviewer. In addition, we have also made extensive revisions to the discussion section in order to align it more directly with the findings in our results section. We agree that our revised article now stands to make an important contribution to the literature.**

Methods :
Line 194-195 : The authors say « . Selected coded segments were translated to English for citation in text ». Does this mean that coding and analysis were conducted in the original interview language by a group of researchers, or one researcher who spoke all of the interview languages ? Please specify who within the research team conducted the analysis, and their background.

**Six total researchers collected the data for this study: one each for Mexico, Kenya, South Africa, and the Philippines and two for India. Researchers were chosen who had experience working and conducting research within each of the countries and were fluent in English as well as the primary language(s) spoken within the country. Interviews were conducted in either English or the primary language of the country, depending on the preference of the key informant being interviewed. When interviews were conducted in a language other than English, selected quotes were translated by the researcher into English for inclusion in the manuscript. Researchers were all PhD candidates or graduates in sociology; 5 were female and one was male. This information has been added to a new section to the methods section of our manuscript titled “Researcher Background.”**

You later mention this in line 551 : « All interview transcripts were coded by the same researcher who conducted the interviews and no double coding was utilized to verify results. » but it should be mentioned during the results, along with the background of the researchers involved in the interviews and analysis, and how the findings across the different sites were ultimately combined.

**As mentioned above, we have added a new section to the methods of our manuscript called “Researcher Background” which provides information on the researchers who conducted the interviews in each country, as well as their background. We have also added clarification to the “Data Analysis” section of our methods about how data was coded and how findings across different sites were ultimately combined.**

Results :
Table 1 : Please harmonize language in the « Target Population » column and use people-centered terminology. For example, the phrase « Bottom third of the population » and « indigent » could be replaced with « low-income groups » or similar. « Transgender » could be « Transgender people ». « IP » should be defined or spelled out in full.

**We have made these changes as requested to Table 1.**

Lines 231 through 400 : In the results section I was expecting to see illustrative quotes that demonstrate the points the authors make in the text, as is the convention in most qualitative research. The authors should read through other qualitative research published in PLOS Global Health and similar journals and see how quotes are incorporated, and seek to similarly introduce illustrative quotes.

**Thank you for this excellent recommendation. We have now added illustrative quotes from our key informants to the results section as recommended.**

The assertions in line 260-263 should be supported with citations or quotes from participants, as right now they seem to be speculation by the authors.

**We have added a quote from a participant to support the assertions in line 260-263.**

Line 301-302 : « Shikilia raised 5 million USD for its cash transfer 302 program, an amount that supported roughly 50,000 households. ». Please specify how long that amount could support 50,000 for.

**We have added information specifying that the funds could support households for 3 months.**

Line 314 through 319: Consider removing the word « valuable » as this is an opinion of the authors. The idea that certain campaigns « played an essential role » also appears to be the opinion of the authors and is not substantiated. The notion that some campaigns contradicted government messaging should be detailed more, to understand specifically what messages the government was giving and how the CSO messaging differed.

**We have removed the terms “valuable” and “essential role,” as recommended. We have also added citations and quotes to provide more detail about how CSO messaging differed from government messaging.**

Line 341 through 344 : The intervention was « most useful » according to whom ?

**The informant from the Development Action Group (DAG) in Cape Town, South Africa indicated that this was the most useful intervention in terms of building responsive links between communities with which DAG was working and municipal and provincial government. We have added a quote to the manuscript to back up this assertion.**

Discussion :
In general, in the discussion section, the authors should take care around the conclusions that can be drawn from information that was gathered only from CSOs and not from the populations the programs sought to benefit. The data collected focuses on the programs implemented by the CSOs and not on the impact felt by the target population. The discussion section is long at at times goes almost an entire paragraph without referring to the direct results of the study. The authors should revise the discussion section, taking care to comment on the results of the study in comparison with existing literature.

**We thank the reviewer for this excellent recommendation, and have worked to revise our discussion section to ensure that it references the findings in our results section, as derived from interviews with our CSO key informants. We have also provided additional citations of existing literature (when appropriate) throughout the discussion section.**

The discussion section could benefit from the addition to more citations to back up the assertions made by the authors. For example, at line 423 the authors state « Many LMIC governments instituted strict lockdowns at the start of the pandemic, following the lead of high-income countries in the Global North, but without regards to the more significant impacts these lockdowns would have on the livelihoods of their populations. » Citations should be provide to support this.

**We have added citations to support this statement as recommended.**

Line 473 : Were the problems of state capacity exposed by the pandemic limited to LMICs ? Were these deficiencies not also observed in HICs ? Consider rephrasing to avoid making this look like an LMIC-only problem.

**We have amended the text to clarify that issues of state capacity were not limited to LMICs.**

Lines 477 through 483, and 495-498 should all be supported with citations from literature.

**We have added citations from the literature to support these sentences.**

The link between discussions of democracy and pandemic preparedness in lines 473 through 493 does not have a substantial tie with the results section. This paragraph could be removed or the link between the results and this discussion point made more clear.

**We have removed the portion of the discussion focused on democracy and pandemic preparedness, as recommended by the reviewer.**

Line 507 : Avoid making generalisations using the word « always ».

**We have removed the word “always” as recommended.**

Line 540 : One key limitation is the lack of community perspective, as to how well these CSO programs responded to actual community needs. Please mention this in the limitations and consider adding as a topic for future research.

**We have added this point to our limitations section and also recommend it as an area for future research.**

Reviewer #2: Congratulations to the author for a well-written and well-thought-out article. In order to highlight CSO's contribution during the pandemic period, such work should be highlighted.

**Thank you, and we agree that this manuscript will make an important contribution to the literature.**

I think a few minor points should be added to the introduction, highlighting the fact that global south cases are underestimated compared to global north cases. So based on that How does underreporting impact the public health response to COVID-19 in the Global South and the allocation of resources?

**This was certainly a significant factor across the global south, especially in Africa and South Asia, as noted in our introduction section. Underreporting in the Global South can be seen as a function of state capacity and politics and often varied by subnational regions, as was the case in India for instance. States with higher capacity and better civil registration systems, underreported to a lesser extent. More than just the allocation of resources, this resulted in delayed public health responses. In addition, "hotspots", especially urban centers, which were reporting higher numbers, received more state attention and CSOs working in these areas perhaps received more donations.**

**In some settings, such as Kenya, the impact of underreporting at the start of the pandemic was likely mitigated by expectations, at the global and national level, that the pandemic would heavily impact densely populated urban areas like Nairobi. As a result, there was a lot of donor funding in the first few months of the pandemic. But underreporting of cases and deaths certainly affected resource availability towards the end of 2020 and afterwards, even in places like Nairobi. As documented in our results, the CSO leaders in Kenya discussed this short-lived nature of financial support.**

**In our introduction, we currently state “The massive underreporting of COVID-19 deaths in the Global South provided a pretense for many national governments, international donors, and international non-governmental humanitarian organizations to not actively intervene, even as it ravaged local communities and economies.” We have added some additional detail to our introduction and discussion section to further highlight this point.**

In the COVID-19 response, what are the differences between the Global South and the Global North in terms of relationships and collaborations between CSOs and other actors (governments, donors, media, communities, etc.)?

**As our research focused only on countries in the Global South, we are not able to directly to compare our results to the experience of CSOs in the Global North. We have noted this in our conclusion as an important area for future research. However, we are able to comment on the relationships between CSOs and other actors, such as government, donors, media, and communities, within the Global South.**

**In general, we noted that the lack of responsiveness of the state to CSO’s demands (due to lack of resources, capacity, or general denial of the effects of the pandemic) led CSOs in the Global South to develop alliances with other local and international actors, creating networks for information-sharing and delivering products, or to find donors to fund their needs. This will likely have impacts for how CSOs relate to the state in the future, and we might see some of these networks survive beyond the pandemic.**

**From the Philippine interviews, for instance, it is quite clear that securing funding became a key driver for CSOs in their interaction with other actors. For example, Community Pantry Ph had a strong connection with private sector through their open donation scheme while Bangsamoro Consortium had a stronger connection with the government as it is its main funding source. Another point to consider is the orientation of the state in addressing the pandemic. The securitized approach in the Philippines generated a disconnect between the state actors and CSO. This drove CSOs to strengthen their relationships with other CSOs and communities. These two points may have taken a different turn in the Global North, though we do not have the data to know for certain.** 

**Similarly, the relationship between the state and civil society in India has been tenuous and strained over the last decade. The pandemic created an opening for some state acknowledgement of the positive role that CSOs were playing in responding to the humanitarian crisis, and in some cases collaboration at a local level. However, these collaborations were restricted to a segment of civil society and were short lived.**

**We have added additional detail to our discussion section covering the points above.**

What are the underlying factors that explain the differences between the Global South and the Global North in terms of the challenges and opportunities they face in the COVID-19 response?

**As mentioned above, our research focused only on countries in the Global South, so we are not able to directly to compare our results to the experience of CSOs in the Global North. We have added this as a recommendation for future research in our Conclusion section.**

How do they ensure adequate representation and diversity of perspectives and experiences? What are the potential biases and limitations of the sampling strategy?

**As described in our Methods section, in order to ensure a representative sample of CSOs within each of the five countries, we selected four areas of CSO diversity, including 1) Geographic scale of operations (i.e. larger national or provincial organizations and smaller community-based groups); 2) Type of organization (i.e. secular, religious, professional/labor, social movements, and consortia/networks; 3) Core covid-related activities (i.e. direct service, livelihoods, advocacy, information; 4) Target population (i.e. general population, specific vulnerable populations). We then performed a mapping exercise where we attempted to identify all CSOs responding to the COVID-19 pandemic in each of the 5 selected countries and categorized them using the areas of diversity described above. Finally, we purposively selected about 10 CSOs per country that represented the full diversity of CSOs across the four categories above, listed in our Table 1. In our Limitations section, we discuss some of the limitations of this methodology, especially that we may have missed some smaller CSOs in our mapping exercise. However, as we were able to include smaller, community based CSOs in each of the five countries, we don’t believe his has impacted our results.**
